# Retraction: Vitamin C and asthma in children: modification of the effect by age, exposure to dampness and the severity of asthma

**DOI:** 10.1186/2045-7022-2-6

**Published:** 2012-03-16

**Authors:** Harri Hemilä, Mohammed Al-Biltagi, Ahmed Abdul Baset

**Affiliations:** 1 Department of Public Health, University of Helsinki, Helsinki, Finland; 2 Department of Paediatrics, Faculty of Medicine, Tanta University, Tanta, Gharbia Governorate, Egypt

## 

We reported that the effect of vitamin C on asthma in Egyptian children was modified by age, exposure to dampness and the severity of asthma [1]. After our paper was published, we found out severe problems in the data set. There were 60 children in the study. The ages were by accident duplicated between the upper and lower halves of the database. Thus, the ages for the first 30 children in the data set were identical and in the same order with the ages for the second set of 30 children. Similar duplication was also found for C-ACT and FEV1 measurements after vitamin C supplementation and for exposure to dampness. This duplication thus directly invalidates the second part of the data set, and thus the reported outcome. We have not been able to sort out the reason for this duplication. The files with the original data are not available any more, making it impossible to reconstruct a valid data set for reanalysis. Therefore we have to retract our paper. The authors deeply regret the inconvenience this has caused to the journal and the scientific community.

## References

[B1] Hemilä H Al-Biltagi M Basset AA Vitamin C and asthma in children: modification of the effect by age, exposure to dampness and the severity of asthma Clinical and Translational Allergy 2011 1 9 10.1186/2045-7022-1-9 22409829 PMC3339402

